# The Periplasmic Chaperone Network of *Campylobacter jejuni*: Evidence that SalC (Cj1289) and PpiD (Cj0694) Are Involved in Maintaining Outer Membrane Integrity

**DOI:** 10.3389/fmicb.2017.00531

**Published:** 2017-03-28

**Authors:** Aidan J. Taylor, Shadi A. I. Zakai, David J. Kelly

**Affiliations:** ^1^Department of Molecular Biology and Biotechnology, The University of SheffieldSheffield, UK; ^2^Faculty of Medicine, King Abdulaziz UniversityJeddah, Saudi Arabia

**Keywords:** *Campylobacter*, outer membrane, periplasmic chaperone, PpiD, SurA, PEB4, VirK, HtrA

## Abstract

The outer membrane (OM) of Gram-negative pathogenic bacteria is a key structure in host–pathogen interactions that contains a plethora of proteins, performing a range of functions including adhesion, nutrient uptake, export of effectors and interaction with innate and adaptive components of the immune system. In addition, the OM can exclude drugs and thus contribute to antimicrobial resistance. The OM of the food-borne pathogen *Campylobacter jejuni* contains porins, adhesins and other virulence factors that must be specifically localized to this membrane, but the protein sorting mechanisms involved are only partially understood. In particular, chaperones are required to ferry OM proteins across the periplasm after they emerge from the Sec translocation system. The SurA-related chaperone PEB4 (Cj0596) is the only protein with a proven role in OM biogenesis and integrity in *C. jejuni*. In this work, we have constructed a set of isogenic deletion mutants in genes encoding both known and predicted chaperones (*cj0596*, *cj0694*, *cj1069*, *cj1228c*, and *cj1289*) using NCTC 11168H as the parental strain. These mutants were characterized using a range of assays to determine effects on growth, agglutination, biofilm formation, membrane permeability and hydrophobicity. We focused on Cj1289 and Cj0694, which our previous work suggested possessed both chaperone and peptidyl-proyl *cis/trans* isomerase (PPIase) domains. Mutants in either *cj1289* or *cj0694* showed growth defects, increased motility, agglutination and biofilm formation and severe OM permeability defects as measured by a lysozyme accessibility assay, that were comparable to those exhibited by the isogenic *peb4* mutant. 2D-gel comparisons showed a general decrease in OM proteins in these mutants. We heterologously overproduced and purified Cj0694 and obtained evidence that this protein was an active PPIase, as judged by its acceleration of the refolding rate of reduced and alkylated ribonuclease T_1_ and that it also possessed holdase-type chaperone activity. Cj0694 is most similar to the PpiD class of chaperones but is unusual in possessing PPIase activity. Taken together, our data show that in addition to PEB4, Cj1289 (SalC; SurA-like chaperone) and Cj0694 (PpiD) are also key proteins involved in OM biogenesis and integrity in *C. jejuni*.

## Introduction

*Campylobacter jejuni* and *Campylobacter coli* are leading causes of human bacterial enteric disease worldwide and these bacteria present a serious ongoing public health and economic problem (O’Brien, 2017). Campylobacters are part of the gut microbiota of many bird and animal species. For *C. jejuni*, undercooked chicken is the main source of human campylobacteriosis and is estimated to be the cause of up to 70% of infections in the UK alone (Sheppard et al., 2009). As a strategically important food-borne pathogen, novel interventions are required to reduce the numbers of campylobacters in the human food-chain. As well as increased bio-security measures, targeted interventions such as poultry vaccines (recently reviewed by Riddle and Guerry, 2016) and the use of specific anti-*Campylobacter* agents or probiotics (Saint-Cyr et al., 2016) on farm have been proposed, but will require identification of appropriate targets and increased knowledge of *C. jejuni* physiology respectively.

The outer membrane (OM) is a structure of the utmost importance in developing such anti-*Campylobacter* strategies. It acts as the interface with the environment and host, and has diverse functions in adhesion, cell signaling, secretion of effectors, host cell damage, and interaction with the immune system (Bos et al., 2007). OM vesicles are also known to be produced by *C. jejuni* (Elmi et al., 2012) and may be a strategy to increase invasion and virulence (Elmi et al., 2012, 2016). Moreover, the OM is an essential permeability barrier (thus affecting antibiotic sensitivity) and a key player in nutrient acquisition, natural competence and biofilm formation. Most of these functions are protein-mediated; in *C. jejuni* the importance of a number of OM proteins (OMPs) have been determined, including porins such as the Major Outer Membrane Porin (MOMP; PorA), the fibronectin binding protein CadF, other adhesins such as PEB1a, CjaA and JlpA and the autotransporter CapA (Rubinchik et al., 2012; Mahdavi et al., 2014; Wu et al., 2016). Highly antigenic OMPs have already been proposed as vaccine candidates, in both chickens and humans (Tribble et al., 2008). However, the mechanism by which OMPs are localized and inserted into the membrane after synthesis in the cytoplasm is still poorly understood in *C. jejuni*.

In Gram-negative bacteria, beta-barrel proteins destined for the OM are translocated through the Sec system in an unfolded state, bound by chaperones in the periplasm, and then presented to an assembly machinery (the “Bam complex”) in the OM itself (Bos et al., 2007). In *Escherichia coli*, two of these periplasmic chaperones that have been well studied are Skp (Seventeen-Kilodalton Protein) and SurA (initially known as a protein required for survival during the stationary phase in *E. coli*) (Bos et al., 2007). Typically, unfolded OMPs bind to the SurA chaperone, but if these substrate proteins fail to interact with SurA, then Skp can bind them (Sklar et al., 2007). The function of these chaperones is to translocate the unfolded OMPs to the OM, where the Bam complex then inserts them. Both *skp* and *surA* mutants are viable; however, a *skp/surA* mutant is synthetically lethal (Rizzitello et al., 2001). This suggests that Skp and SurA are functionally related and they work by similar mechanisms for chaperone activity. Other periplasmic chaperone-like proteins in *E. coli* including PpiA, PpiD and FkpA have been identified, which may bind a wider range of client proteins than just OMPs. PpiD is thought to aid in the early periplasmic folding of a diverse array of newly translocated proteins emerging from the Sec translocon, but may not be specifically involved in the maturation of OMPs (Matern et al., 2010). SurA and PpiD contain domains homologous to the small peptidyl-prolyl *cis/trans* isomerase (PPIase) parvulin; an enzyme required for the *cis/trans* isomerisation of proline residues (Stymest and Klappa, 2008). The role of PPIase domains in such chaperones is not always clear, as the *E. coli* PpiD parvulin-like domain is catalytically inactive (Weininger et al., 2010) as is one of the two parvulin domains in SurA itself. Another potential chaperone, VirK, is a 37 kDa periplasmic protein that may have a role in autotransporter assembly and toxin export in *E. coli* (Tapia-Pastrana et al., 2012). Finally, HtrA (DegP) is a chaperone and proteolytic enzyme that degrades unfolded proteins in the periplasm (Ge et al., 2014).

*Campylobacter jejuni* possesses a network of both proven and putative periplasmic chaperones, but the roles of only a few of these are clear. A homolog of the HtrA protein has been shown to be important for protecting against heat and oxidative stress and possesses both chaperone and serine protease activities (Brøndsted et al., 2005; Baek et al., 2011). It is also a secreted protein that plays a role in host cell invasion by cleavage of *E*-cadherin (Hoy et al., 2012). A *C. jejuni* VirK homolog has been identified as a virulence factor in mouse colonization and involved in resistance to antimicrobial peptides, but unlike the situation in *E. coli*, seemed to be localized at the cytoplasmic side of the inner membrane (Novik et al., 2009). A highly conserved protein, PEB4 (Cj0596), has weak sequence similarity to SurA and from mutant studies has been implicated in the assembly of several OMPs in *C. jejuni* (Asakura et al., 2007; Rathbun et al., 2009). Asakura et al. (2007) showed that a *peb4* mutant in *C. jejuni* strain NCTC 11168 had reduced biofilm formation, adhered less well to INT407 cells than the wild-type and exhibited a lower level and duration of intestinal colonization of a mouse model. Rathbun et al. (2009) also found that a *peb4* mutant generated in strain 81–176 had a reduced ability to colonize mice. These data therefore support a direct correlation between changes in OM protein assembly and virulence in *C. jejuni*.

Kale et al. (2011) elucidated the crystal structure of PEB4. At a resolution of 2.2 Å, its structure reveals a dimeric organization with SurA-like chaperone and PPIase domains. However, unlike SurA, the overall fold of PEB4 is distinct. A large chaperone domain comprising the *N*- and *C*-terminal regions of the protein is linked to a second domain that has a standard PPIase fold. The chaperone domain is closely related to that of SurA but is different in the way helices from both domains interlock to form a domain-swapped structure (Kale et al., 2011). The PPIase domain in PEB4 is active when assayed using denatured ribonuclease T_1_, which showed a significant PEB4-dependent acceleration in proline isomerization-limited refolding and PEB4 also strongly inhibits the aggregation of renaturing model proteins like rhodanese. It was therefore suggested that PEB4 is a holdase-type chaperone whose function is to inhibit protein folding and aggregation prior to delivery of the client protein to the Bam complex (Kale et al., 2011).

Using the SurA chaperone domain in structure prediction searches, two other related chaperones have been identified in *C. jejuni*; Cj1289 and Cj0694 (Kale et al., 2011). Unlike PEB4, the Cj1289 crystal structure at 2.4 Å did not show a domain-swapped structure, which makes it much more similar to SurA itself. However, it only has one parvulin-like PPIase domain instead of the two found in SurA. This domain was active in increasing the refolding rate of ribonuclease T_1_. Nevertheless, purified Cj1289 did not prevent rhodanese refolding and aggregation (Kale et al., 2011), suggesting that Cj1289 may chaperone specific *C. jejuni* substrates. Cj0694 has weak sequence similarity to the *E. coli* PpiD protein discussed above. This, combined with a similar domain organization and predicted N-terminal membrane anchored region suggest a similar role for Cj0694 in *C. jejuni* as for PpiD in *E. coli*, although Kale et al. (2011) could not obtain a soluble form of recombinant Cj0694 for biochemical or structural studies.

In this study, we have constructed isogenic deletion mutants in all of the proven and putative periplasmic chaperones in *C. jejuni* NCTC 11168H and compared the overall impact on OM integrity by determining their phenotypes with respect to growth and OM permeability properties. We have focused on the poorly characterized Cj1289 (which we designate SalC; SurA-like chaperone) and Cj0694 (designated PpiD) by analyzing the OM and periplasmic protein profiles of the cognate mutants and we show that the purified Cj0694 protein possesses both PPIase and chaperone activity.

## Materials and Methods

### Bacterial Strains and Culture Conditions

*Campylobacter jejuni* NCTC 11168H cultures were maintained on Columbia agar base plus 5% v/v defibrinated horse blood at 42°C in microaerobic conditions [10% (v/v) O_2_, 5 % (v/v) CO_2_, and 85 % (v/v) N_2_] generated in a MACS-VA500 Microaerobic Workstation (Don Whitley Scientific Ltd, Shipley, UK). Liquid cultures were routinely grown at 42°C in Brucella broth base (Sigma) plus 1% w/v tryptone and 20 mM L-serine (BTS broth) under standard microaerobic conditions with continuous orbital shaking at 140 rpm. Overnight starter cultures were allowed to grow from fresh 18 h old cells grown on plates prior to inoculation of larger cultures. Plates and broth cultures used for *C. jejuni* growth routinely contained amphotericin B and vancomycin at 10 μg ml^-1^, with kanamycin and apramycin at 50 μg ml^-1^ where appropriate. *E. coli* strains were cultured aerobically at 37°C on LB agar or in LB broth (Melford laboratories, UK) with orbital shaking (225–250 rpm), with selective antibiotics carbenicillin, kanamycin or apramycin at 50 μg ml^-1^, where appropriate.

### Cloning, Mutagenesis and Complementation Vector Construction

Putative chaperone genes were inactivated by deletion and insertion of a kanamycin resistance cassette into the reading frame by double homologous cross-over of the mutant allele from a pGEM-3ZF vector containing the kanamycin resistance cassette flanked by ∼500 bp upstream and downstream of the target gene. Mutant vectors were created using the Gibson isothermal assembly method as described by Gibson et al. (2009). Briefly, gene flanking regions were amplified from *C. jejuni* 11168H genomic DNA using primers with adapters homologous to either the kanamycin cassette amplified from pJMK30, or the ends of HincII linearised pGEM-3ZF (Supplementary Table 1). The isothermal assembly reaction specifically anneals all four fragments together to yield the mutant plasmid. Correct constructs were confirmed by automated DNA sequencing. Wild-type *C. jejuni* 11168H was transformed by electroporation and mutants selected on blood agar for kanamycin resistance. Correct insertion of the kanamycin cassette into the genome was confirmed by PCR. Complementation vectors were based on the pRRA system as described by Cameron and Gaynor (2014). Briefly, *cj0694* and *cj1289* were amplified from *C. jejuni* 11168H genomic DNA, including their promoter region, with MfeI and XbaI restriction site adaptors. *cj0694* and *cj1289* both contained an internal restriction site, requiring partial digestion and gel purification of the desired full length insert. Digested inserts were ligated with similarly digested, rSAP phosphatase treated pRRA and transformed into *E. coli* DH5α under apramycin selection. Putative clones were screened by PCR and purified plasmids further screened to ensure insertion of the full length gene. HΔ*cj0694* and HΔ*cj1289* mutants were transformed by electroporation with their respective complementation vector and clones selected for apramycin resistance. Correct insertion of the expression cassette into the genome was confirmed by PCR. All primers are listed in Supplementary Table 1.

### Lysozyme Accessibility Assay

Cells from 12 h old broth cultures were washed twice in 20 mM sodium phosphate buffer, pH 7.4, by repeated centrifugation (12,000 × g, 25°C, 5 min) and resuspension of the cell pellet, then adjusted to an optical density at 600 nm of ∼0.8. Nine hundred and ten microliter of cells were mixed by inversion with 50 μl of freshly prepared 0.1 mg ml^-1^ lysozyme from chicken egg white (Sigma) in phosphate buffer and the absorbance at 600 nm monitored for 20 s to provide a background drift rate. Forty microliter of 62.5 mM sodium deoxycholate in phosphate buffer was added, mixed by inversion and the measurement continued for a further 60 s. The rate of cell lysis was determined by subtracting the drift rate from the rate of decrease in absorbance after the addition of deoxycholate. The *cj0694* and *cj1289* mutants have a very compromised OM and were found to show significant lysis even in the absence of deoxycholate. Therefore, for complementation data with these mutants, the susceptibility to lysozyme was determined by subtracting the drift rate (without lysozyme) from the rate of decrease in absorbance after the addition of lysozyme. All experiments were performed in triplicate in a Shimadzu UV-2401 spectrophotometer at 25°C.

### Auto-Agglutination (AAG) Assay

Cells from 12 h old broth cultures were washed twice in 20 mM sodium phosphate buffer, pH 7.4, by repeated centrifugation (12,000 *g*, 25 C, 5 min) and resuspension of the cell pellet, then adjusted to an optical density at 600 nm of exactly 0.40. Six milliliter of cells were transferred to glass tubes (18 mm diameter) in duplicate and left undisturbed at room temperature. One milliliter samples were carefully taken from the top of each aliquot at various time points and the optical density at 600 nm recorded. The experiments were performed in duplicate.

### Cell Surface Hydrophobicity Assay

Cells from 12 h old broth cultures were washed twice in 20 mM sodium phosphate buffer, pH 7.4, by repeated centrifugation (12,000 *g*, 25 C, 5 min) and resuspension of the cell pellet, then adjusted to an optical density at 600 nm of ∼0.8. 3 ml of cells were mixed with 1 ml of *n*-hexadecane by vortexing for 30 s, then left undisturbed for 20 min before 1 ml of the aqueous phase cells was carefully removed and the optical absorbance at 600 nm read. Controls without hexadecane were performed to account for autoagglutination and cell lysis as a result of vortexing. The experiments were performed in triplicate, with H-values determined from optical absorbances at 600 nm by the equation:

((control sample−test sample)/control sample)*100

### Motility Assay

Bacterial motility was determined by seeding semi-solid (0.4% w/v agar) brain-heart infusion (BHI) plates with 5 μl of cells set to an optical absorbance at 600 nm of 1.0 in BTS broth and measuring the diameter of the swarm rings after 48 h. Plates contained 150 μM triphenyltetrazolium chloride (TTC) to visualize growth and improve the accuracy of growth diameter measurement.

### Biofilm Assay

Static biofilm formation assays were performed in 96 well flat bottom plates containing one strain per row (12 replicates). Cells from 12 h old broth cultures were adjusted to an optical density at 600 nm of exactly 0.1 in BTS broth and 200 μl of each culture added to the appropriate wells. Plates were incubated without shaking under standard microaerobic conditions for 24 h. Planktonic cells were carefully pipetted off and 200 μl of 1% (w/v) crystal violet in 95% (v/v) ethanol added to each well. After 5 min incubation, the crystal violet stain was pipetted off and wells gently washed twice with 400 μl dH_2_O. The remaining biofilm-bound dye was resuspended in 200 μl of ethanol:acetone (4:1) by agitation of plates at room temperature for 20 min. The optical absorbance at 600 nm of the crystal violet was measured in a Victor2 1420 Multilabel Counter plate scanner (Perkin Elmer, USA). BTS broth controls were used as the blank. All data was divided by the average of the wild-type values to give final data as a ratio of wild-type biofilm formation.

### Preparation of *C. jejuni* Periplasm and Outer Membrane Fractions

*Campylobacter jejuni* strains were grown in 0.5–1.0 L culture volumes until early stationary phase and an OD600 nm of ∼1.0–1.2 (∼16 h) in standard microaerobic conditions. Cells were harvested by centrifugation (7,155 *× g*, 15 min and 4°C), gently resuspended in 20 mL STE buffer [20% (w/v) sucrose, 30 mM Tris-HCl pH 8.0 and 1 mM EDTA] and incubated at room temperature with gentle shaking for 30 min. Then, cells were harvested by centrifugation (11,180 *× g*, 10 min and 4°C) and the supernatant was discarded. The pellets were resuspended in ice-cold 10 mM Tris-HCl pH 8.0 buffer and incubated with gentle shaking at 4°C for 1 h, then centrifuged (15,000 *× g*, 25 min and 4°C). The resulting osmotic shock supernatant containing the periplasmic proteins was stored at – 20°C. The pellet was then used to isolate the OM fraction as follows. The pellets were resuspended in 10 mM HEPES buffer pH 7.4 and sonicated with ice cooling for 6 × 15 s pulses at a frequency of 10 microns using a Soniprep 150 ultrasonic disintegrator (SANYO, Japan). Unbroken cells and cell debris were removed by centrifugation (27,167 *× g*, 30 min and 4°C). The supernatant was transferred to pre-chilled ultra-centrifuge tubes, and the inner and the OMs were pelleted by ultra-centrifugation (100,000 *× g*, 60 min and 4°C). The red pellet containing both inner and OM was resuspended in 2 mL of 10 mM HEPES buffer pH 7.4. The inner membrane was dissolved by addition of 2 mL of 2% sarkosyl (Sodium *N*-lauryl sarcosinate) dissolved in 10 mM HEPES buffer pH 7.4 and incubated at 37°C for 30 min. A further centrifugation (48,297 *× g*, 30 min and 4°C) was carried out to isolate the OM. The supernatant containing the solubilised inner membrane was carefully and fully removed and the OM pellet was washed three times in HEPES buffer prior to being homogenized in 0.5 – 1 mL of 10 mM HEPES buffer pH 7.4 and stored at -20°C.

### 2D-PAGE and Mass Spectrometry

The OM and periplasmic fractions from independent replicate cultures were analyzed by 2D-PAGE using the methods described previously (Hitchcock et al., 2010). Samples were solubilised in rehydration lysis buffer (RHB; 7 M urea, 2 M thiourea, 2% CHAPS). The first dimension was run on 18 cm 3-11NL Immobiline DryStrips (GE healthcare, UK). Following overnight rehydration, IEF was performed for 80 kVh at 20°C over 24 h using the pHaser system (Genomic Solutions, UK). The focussed strips were treated as described previously (Leon-Kempis Mdel et al., 2006). The second dimension used 4-12% Novex precast gels (Thermo Fisher). Proteins were stained by SYPRO-Ruby (Bio-Rad, UK) and the gels imaged using a Pharos FX+ Molecular Imager with Quantity One imaging software (BioRad, UK). For comparisons, the digital images were differentially colored and overlayed. For protein identification, selected spots were excised from the gel using the ProPick excision robot (Genomic Solutions, UK) and trypsin digested. Tryptic digests were analyzed using a Reflex III MALDI-TOF instrument (Bruker, UK). Proteins were identified by the Protein Mass Fingerprint technique using the MASCOT search tool (Matrix Science^1^).

### Immunoblotting

To assess periplasmic contamination of OM fractions, immunoblotting to detect the periplasmic protein MfrA (Guccione et al., 2010) was performed. Protein samples were separated on 10% SDS-PAGE gels and transferred to a nitrocellulose membrane (Hybond-C Extra, Amersham Biosciences) using a Mini Trans-Blot Electrophoretic Cell (Bio-Rad, USA). The transfer of protein was carried out at a constant current of 400 mA for 60 min at 4°C in ice cold transfer buffer [25 mM Tris, 190 mM glycine, 20% *(v/v)* methanol]. All transfers were performed at RT with constant stirring. 1X TBS-T buffer [25 mM Tris-HCl pH 7.4, 137 mM NaCl, 2.7 mM KCl, 0.1% *(v/v)* Tween 20] was used as a base for washing the nitrocellulose membrane (20, 10, and 5 min). 1X PBS-T was used as a base for blocking the membrane [standard phosphate buffered saline plus 0.1% *(v/v)* Tween 20 plus 5% *(w/v)* dried skimmed milk incubated at least 1 h with gentle shaking at RT or overnight at 4°C]. After blocking, the membrane was washed with 1X TBS-T buffer with gentle shaking for 20 min and twice for 5 min. Primary anti-MfrA polyclonal antibody raised in rat (Guccione et al., 2010) was diluted in 1X TBS-T buffer (1:2000) and incubated with the membrane for 1 h with gentle shaking. The membrane was washed with 1X TBS-T for 10 min and twice for 5 min. Secondary antibody (peroxidase-linked Anti-Rat IgG [whole molecule], Sigma) was diluted (1:2000) in TBS-T and incubated with the membrane for another hour. Washing of the membrane in 1X TBS-T was for 10 min and three times for 5 min. Visualization of antibody binding was performed by enhanced chemi-luminescence (ECL Kit, GE Healthcare) and exposure to x-ray film.

### Overproduction and Purification of His-tagged Cj0694

Over-production of Cj0694 was performed by cloning the *cj0694* coding sequence minus the first 102 bp encoding the predicted transmembrane anchor (residues 1–34) into the l-arabinose inducible pBAD/His B vector (Invitrogen), which fuses a 6xHis tag to the *N*-terminal end of the protein. The primers 0694-OEF-pBAD and 0694-OER-pBAD (Supplementary Table 1) were used with *C. jejuni* genomic DNA as template and the ∼1.4 kb amplicon cloned into the *Xho*I and *Eco*RI sites of the vector to give pBAD0694, which was transformed into *E. coli* TOP10 cells. *E. coli* TOP10 (pBAD0694) was grown in LB broth containing 50 μg mL^-1^ carbenicillin at 37°C until OD_600_ ∼0.6. Over-expression was induced by addition of 0.02% w/v l-arabinose and cultures were incubated at 37°C with shaking at 250 rpm for 24 h. Cells were harvested by centrifugation and cell-free extracts prepared by sonication. Ice-cold cell-free extracts were fractionated on a 5 ml HisTrap^TM^ column (GE Healthcare, UK). Proteins were bound to the column in binding buffer (20 mM Tris-HCl pH 8.0, 500 mM NaCl, 20 mM imidazole) and eluted from the resin over 10 column volumes with a linear gradient of 20–500 mM imidazole in the same buffer. Protein-containing fractions were then pooled, concentrated using a Vivaspin 20 column to a final volume of 2–5 mL, and buffer exchanged into 50 mM Tris-HCl pH 8.0 before SDS-PAGE analysis. These samples were further purified by loading onto a 5 ml HiTrap^TM^ DEAE FF column (GE Healthcare, UK) and proteins were eluted in the same buffer over 10 column volumes with a linear gradient of 0–1 M NaCl. Protein-containing fractions were analyzed by SDS-PAGE. *N*-terminal sequencing, by automated Edman degradation, was performed by Dr. Arthur Moir (Department of Molecular Biology and Biotechnology, University of Sheffield).

### PPIase Assay

The wild-type ribonuclease T_1_ from *Aspergillus oryzae* (Sigma) was used to evaluate proline isomerisation-limited protein folding as described by Scholz et al. (1997). Disulphide reduced and *S*-carboxymethylated RNase T_1_ (RCM-RNase T_1_) was prepared following the method developed by Mücke and Schmid (1992). Firstly, 0.036 μmol of RNase T_1_ was incubated in 275 μL denaturation buffer (6 M guanidine-hydrochloride [GdnHCl] and 2 mM EDTA in 0.2 M Tris-HCl, pH 8.7) for 2 h at 25°C. Then, 30 μL of the reducing buffer (20 mM dithiothreitol [DTT], 6 M GdnHCl, and 2 mM EDTA in 0.2 M Tris-HCl, pH 8.7) was added and protein reduction under argon was carried out at 25°C for 2 h. Next, 60 μL of the carboxymethylation buffer (0.6 M iodoacetate in 0.2 M Tris-HCl, pH 7.5) was added and the sample was incubated in the dark for 5 min at 25°C. This step was essential to ‘cap’ the cysteine residues and prevent the formation of two disulphide bonds during the denaturation process. Finally, 100 μL of 0.5 M reduced glutathione in 0.2 M Tris-HCl, pH 7.5, was added to stop the reaction. The RCM-RNase T_1_ was separated from the reagents by dialysis against 10 mM Tris-HCl pH 8.0 at 4°C overnight. The refolding of wild-type RCM-RNase T_1_ (which is rate-limited by the prolyl *cis-trans* isomerisation of Pro39 and Pro55 (Mücke and Schmid, 1992), was followed by monitoring the changes in the intrinsic tryptophan fluorescence. Refolding was initiated by a 50-fold dilution of the unfolded protein (stored in the absence of NaCl) to a final concentration of 1.2 μM in a buffer containing 0.1 M sodium acetate, pH 5.0, and 4 M NaCl. Changes in the steady-state Trp59 fluorescence were measured at 320 nm (10 nm bandwidth) with excitation at 268 nm (2.5 nm bandwidth) using a Varian Cary Eclipse spectrofluorimeter with the temperature maintained at 15°C. PEB4 (0.5 μM final concentration) or Cj0694 (0.25 or 0.5 μM final concentration) were added to the RCM-RNase T_1_ (final concentration of 0.5 μM). Immediately following dilution into the high salt buffer, the fluorimeter was zeroed and the increase in fluorescence intensity recorded. PEB4 was over-produced and purified for comparative assays as described previously (Kale et al., 2011).

### Cj0694 Chaperone Assay

Chaperone activity was demonstrated by measuring the effect of Cj0694 on the aggregation of the model proteins lysozyme and rhodanese during renaturation, following their denaturation with guanidine-HCl. Unfolding and refolding of rhodanese and lysozyme was carried out as previously described (Ideno et al., 2000). Typically, 30 μM pure rhodanese or lysozyme (Sigma) was first denatured for 2 h at 25°C in 50 mM Tris-HCl, pH 7.8, containing 6 M guanidine-HCl and 20 mM DTT. Renaturation was initiated by a 60-fold dilution in 50 mM Tris-HCl, pH 7.8, to reach a final concentration of 1.0 μM of rhodanese or lysozyme, in the absence or presence of Cj0694 (1.0–5.0 μM) or bovine serum albumin (1.0 μM) as a negative control. The reactions were maintained at 25°C. The light scattering resulting from the formation of protein aggregates was measured by increase in the absorbance at 320 nm in a Shimadzu UV-2401PC spectrophotometer.

## Results

### Physiological Phenotypes of Mutants in Candidate Periplasmic Chaperone Genes

**FIGURE 1:**
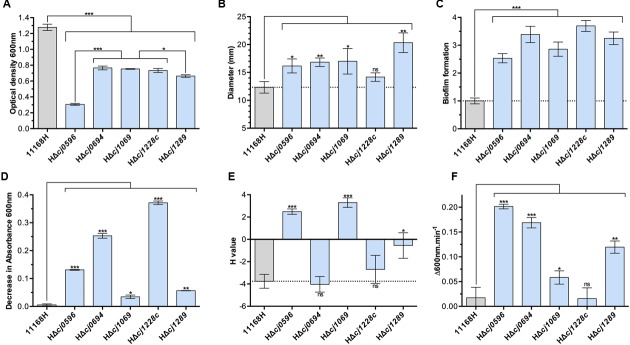
**Physiological phenotypes of chaperone mutants (blue bars) and isogenic parent strain *C. jejuni* 11168H (gray bars). (A)** Growth measured as cell density at 600 nm at 12 h post-inoculation in BTS broth under standard microaerobic conditions. **(B)** Motility of strains determined by point inoculation of semi-solid agar plates and measuring the diameter of growth after 48 h. **(C)** Biofilm formation in BTS broth in 96-well plates after 24 h determined by crystal violet staining. **(D)** Autoagglutination (AAG) activity determined by the decrease in absorbance of cells in the aqueous phase of static cultures. **(E)** Cell surface hydrophobicity determined by the change in cell absorbance in the aqueous phase after mixing cells with the alkane hydrocarbon hexadecane. **(F)** Susceptibility to cell lysis by lysozyme digestion in the presence of 2.5 mM deoxycholate. Student *t*-test *P*-values are displayed as ^∗^ < 0.05, ^∗∗^ < 0.01, ^∗∗∗^ < 0.001.

The effects of removal of Cj0596 (PEB4), Cj1069 (VirK) and Cj1228 (HtrA) have been previously studied in mutants made in different parental wild-type backgrounds (Novik et al., 2009; Rathbun et al., 2009; Boehm et al., 2015). Here, we sought to compare the phenotypes of these mutants with those in genes encoding the putative chaperones Cj0694 and Cj1289, which have not previously been characterized, in a single parental strain background. We therefore constructed a set of isogenic deletion-insertion mutants in *cj0596*, *cj0694*, *cj1069*, *cj1228c*, and *cj1289* in *C. jejuni* NCTC 11168H, a well characterized motile variant of the NCTC 11168 reference strain (Karlyshev et al., 2002), so that we could determine the physiological phenotypes of all five mutants using a range of assays relating to growth, cell surface properties and OM integrity (**Figures 1A–F**).

Deletion of either *cj0694* or *cj1289* was not lethal, but these and the other mutants showed a pronounced growth defect under the conditions tested (microaerobic conditions in complex media), particularly HΔ*cj0596* (*peb4*), consistent with a pleotropic cell envelope defect (**Figure 1A**). Motility was enhanced in all mutants except *htrA* (**Figure 1B**) and biofilm formation was increased in all mutants (**Figure 1C**). These data are consistent with results reported previously for a *peb4* mutant in strain 81–176, where motility and biofilm formation were both enhanced (Rathbun and Thompson, 2009; Rathbun et al., 2009).

Cell surface characteristics were assayed by autoagglutination ability (**Figure 1D**) and cell surface hydrophobicity (**Figure 1E**). All mutants showed increased autoagglutination, but to widely varying degrees; this was most pronounded for the *peb4, cj0694* and *htrA* mutants. Interestingly, the *peb4* and *virK* mutants showed a strongly increased cell surface hydrophobicity, whereas the *cj0694* mutant was unchanged and the *cj1289* mutant showed only a mildly increased hydrophobicity compared to the wild-type. There was no apparent link between autoagglutination and hydrophobicity, suggesting specific OM and/or secreted proteins that are absent, or present at a reduced level, in some mutants may be important in these processes.

Finally, OM integrity was assayed by the susceptibility of cells to lysis by lysozyme. Lysozyme can lyse cells by digestion of the peptidoglycan layer, but this protein (14 kDa) can only access the periplasm when the OM is compromised. Therefore, the rate of cell lysis by lysozyme, especially in the presence of a membrane perturbing compound, (we used the major physiological bile salt sodium deoxycholate as an enhancer), can be interpreted as a function of OM integrity, independent of the inner membrane (**Figure 1F**). The chaperone roles of HtrA and VirK are not thought to be specifically related to OM proteins and so it was expected that their removal from the cells should not lead to an OM integrity defect. In keeping with this, the *htrA* mutant showed no increased susceptibility to lysis compared to wild-type, and the *virK* mutant displayed only a weak phenotype. However, each of the *peb4*, HΔ*cj0694* and HΔ*cj1289* mutants displayed evidence of highly compromised OM integrity (**Figure 1F**), demonstrating the importance of these putative chaperones in OM structure.

### Complementation of HΔ*cj0694* and HΔ*cj1289* Restores OM Integrity

**FIGURE 2:**
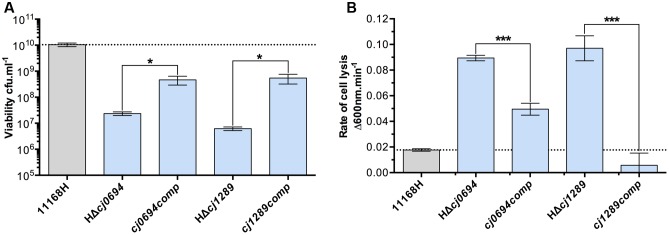
**Genetic complementation of HΔ*cj0694* and HΔ*cj1289* strains. (A)** Viability of mid-log BTS broth cultures diluted to an optical density at 600 nm of exactly 0.1, as determined by serial dilution and colony counts. **(B)** OM integrity as determined by susceptibility to cell lysis by lysozyme digestion. Student *t*-test *P*-values are displayed as ^∗^ < 0.05, ^∗∗∗^ < 0.001.

Complemented strains of HΔ*cj0694* and HΔ*cj1289* were made using the pRRA vector system, as described in Section “Materials and Methods,” with the respective genes expressed from their native promoter (Cameron and Gaynor, 2014). Complementation vectors were transformed into their respective mutant in the 11168H background by electroporation and clones selected for dual kanamycin and apramycin resistance. Genomic DNA was screened by PCR to confirm correct genomic insertion of the target gene into the 16S/28S rRNA locus. Complemented strains showed significant restoration of their growth defect, as measured by increased cell viability under standard microaerobic conditions, and partial to complete restoration of OM integrity measured by lysozyme sensitivity (**Figure 2**). This confirms the phenotypes described for HΔ*cj0694* and HΔ*cj1289* strains are specifically due to their deletion, and supports their role as periplasmic chaperones that, when deleted, significantly alter the structure and integrity of the OM in *C. jejuni*.

### Changes in OM and Periplasmic Proteins in *cj0694* and *cj1289* Mutants

**FIGURE 3:**
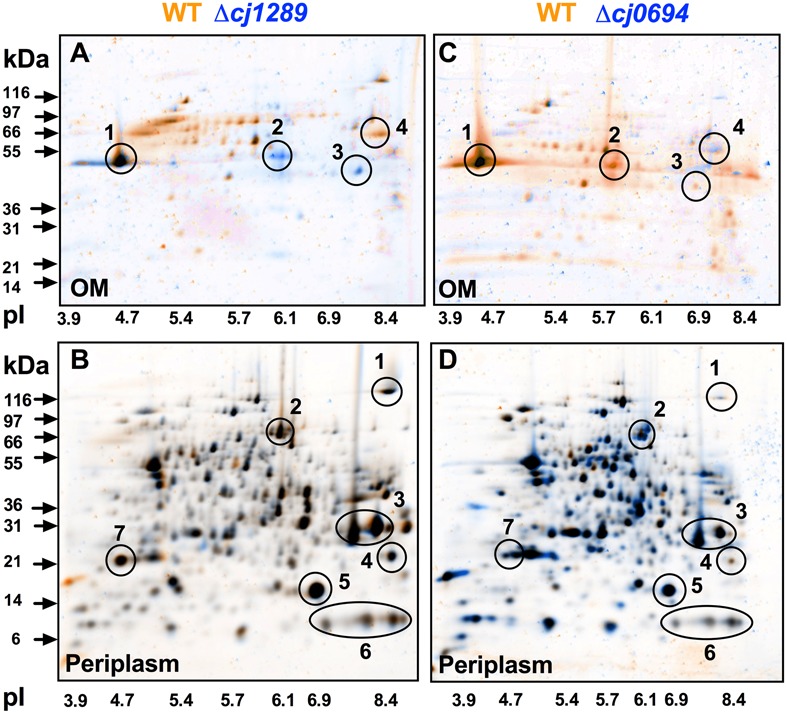
**2D-PAGE analysis of the outer membrane and periplasmic proteins of the wild-type (colored orange) and *cj0694* and *cj1289* mutants (colored blue).** Protein samples were prepared as described in Section “Materials and Methods” and separately resolved by 2D-PAGE. Orange spots represent proteins found in the wild-type fractions only, and absent in the mutant fractions. Blue spots represent proteins found in mutant fractions only, and absent in the wild-type fractions. Black spots represent proteins found in both the wild-type and mutant fractions. In **(A,C)**, the circled numbered protein spots in the 2D-gels of the OMs were identified by mass spectrometry analysis. These are: 1, MOMP (PorA); 2, Cj0964 (Mascot score 88); 3, Cj0112 (TolB, Mascot score 2115); 4, Cj1228 (HtrA, Mascot score 2056). In **(B,D)**, the circled numbered protein spots in the periplasms were correlated with those published in our previous study (Hitchcock et al., 2010). These are: 1, TorA; 2, MfrA; 3, Peb1A; 4, Cj0998; 5, Cj0715; 6, Cj1153; 7, Cj1659 (p19 protein).

Outer membrane and periplasmic fractions of the wild-type and isogenic *cj1289* and *cj0694* mutants were obtained as described in Section “Materials and Methods,” in order to identify global changes in protein abundance and potentially identify any client OMPs that are dependent on Cj1289 or Cj0694 for their maturation and assembly in the OM. An analysis by 2D-PAGE was carried out, with the gels stained with SYPRO-Ruby. The images of the WT and mutant periplasm and OM fractions were digitally overlaid using either orange or blue coloring of the protein spots so that differences in protein abundance could be more easily observed; proteins with the same abundance appear black in such overlays (**Figure 3**). Overlaying the 2D-gels of the OMs (**Figures 3A,C**) of the wild-type (stained in orange) and the *Δcj1289* or *cj0694* mutants (stained in blue) suggested an overall reduction in OMP abundance in both of these mutants compared to wild-type, as many of the spots detected showed up as more orange in the overlays (i.e., more abundant in WT). The prominent major-outer membrane porin (MOMP) appeared black, suggesting it was similar in abundance in wild-type and both mutants. However, two proteins stand out as blue in the overlay of the *Δcj1289* mutant OM but orange in the *Δcj0694* overlay. These were identified as Cj0112 (TolB) a periplasmic component of the OM Tol transport system and Cj0964, a putative periplasmic protein, which may therefore not be *bona fide* OM-associated proteins. The purity of the OM fractions was therefore assessed by immunoblotting using anti-MfrA, raised against the very abundant periplasmic subunit of the methylmenaquinol:fumarate reductase (MfrA) in *C. jejuni* (Guccione et al., 2010) (Supplementary Figure 1). This showed that the ∼65 kDa MfrA protein is present exclusively in the periplasmic but not in the OM fractions of the wild-type and *cj0694* mutant, but with evidence of a faint band of the same size present in the *cj1289* OM fraction. Thus, Cj0112 and Cj0964 are most likely to be contaminating periplasmic proteins especially in the *Δcj1289* OM fraction. Overlaying the 2D-gels of the periplasmic fractions of the wild-type (stained in orange) and the *Δcj1289* mutant (stained in blue) showed very little alteration in the protein profiles or abundances, with most of the proteins appearing black (**Figure 3B**). Comparison of the 2D-gels of the periplasms of the wild-type and the *Δcj0694* mutant showed there were more bluish spots and thus more accumulation of proteins in the periplasm of this mutant (**Figure 3D**). This might be expected if some client OM proteins of Cj0694 are now mislocalised to the periplasm. Overall, the general reduction in OMPs in the OM fractions of these mutants is consistent with a role for both Cj0694 and Cj1289 in OMP biogenesis.

### Over-Production and Purification of Cj0694

**FIGURE 4:**
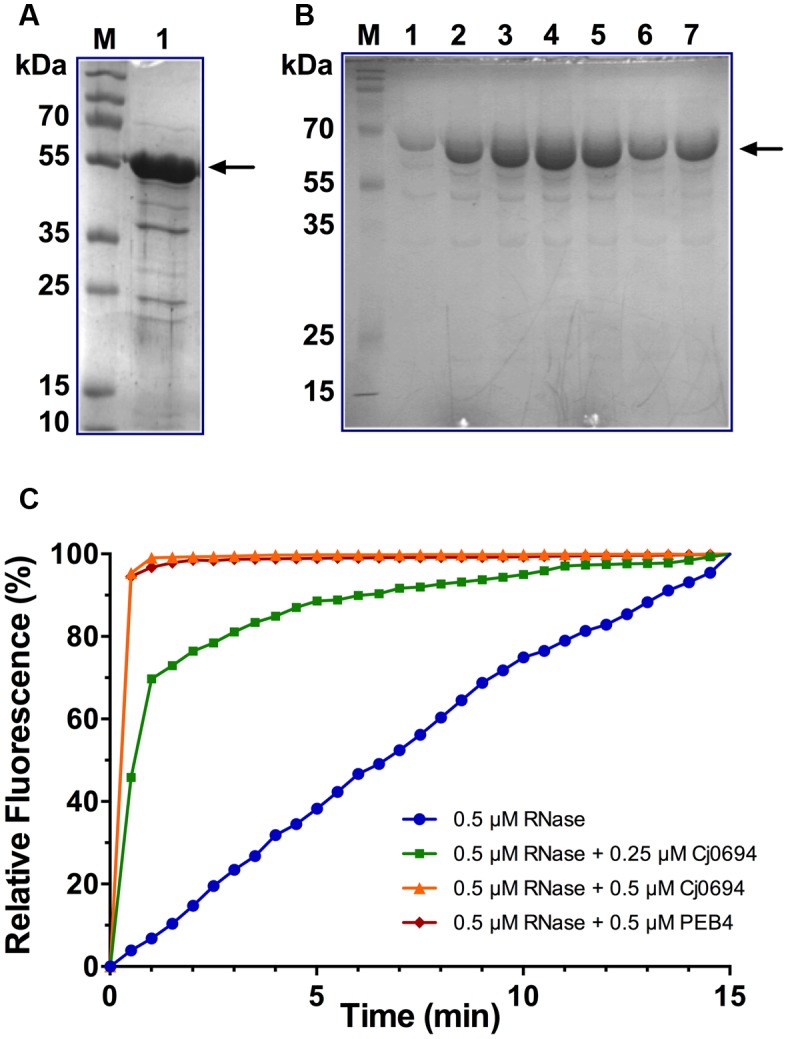
**Overproduction, purification and PPIase activity of Cj0694. (A)** Lane 1; Cj0694 lacking the N-terminal membrane anchor and with a C-terminal his-tag initially purified by Ni-NTA affinity chromatography (monomer molecular weight 54.8 kDa). Lane M; molecular weight markers. **(B)** Further purification of Cj694 by DEAE anion-exchange chromatography with elution from 0 to 1 M NaCl. Lanes 1–7 are samples taken across the UV-absorbing peak eluted from the column and show a single major band on SDS-PAGE after staining with Coomassie Blue. Note that the migration of the protein is affected by the salt present in the elution buffer and the apparent molecular weight is higher than in **(A)**. Lane M; molecular weight markers. **(C)** PPIase activity of Cj0694 demonstrated by refolding of RCM-RNaseT_1_ in the presence of 4 M NaCl (see Materials and Methods) either without or with the addition of purified Cj0694 as shown. The purified periplasmic chaperone PEB4 was used as a positive control. The fluorimeter was set to zero at the time of dilution, so that the increase in fluorescence results from the uncatalyzed (blue progress curve) or chaperone catalyzed (green, orange, and red progress curves) refolding process. Results shown are a single representative experiment.

Although Kale et al. (2011) investigated the PPIase and chaperone properties of Cj1289, these activities could not be determined for Cj0694 due to problems with heterologous expression of the protein in a pET vector system. Here, the *cj0694* gene was cloned and successfully over-expressed in the pBAD vector system as described in Section “Materials and Methods.” The over-produced Cj0694 recombinant protein lacking the N-terminal membrane anchor and containing a hexahistidine tag was initially purified by Ni-NTA affinity chromatography. This resulted in a considerable enrichment, although the protein was not pure (**Figure 4A**). Anion-exchange chromatography using a DEAE-sepharose column resulted in significant further purification, as judged by Coomassie blue staining (**Figure 4B**; note that the presence of salt in the column elution buffer slows the migration of the protein so it appears larger than in **Figure 4A**). The purified protein was stable for at least a week at 4°C. *N*-terminal sequencing confirmed the expected sequence MGGSHHHH. The protein ran as a monomer on a calibrated gel filtration column, with an estimated native molecular weight of 54.8 kDa.

### Cj0694 has PPIase Activity and Accelerates the Refolding of RCM-RNase T_1_

Our previous bioinformatics analysis revealed that Cj0694 is a homolog of PpiD in *E. coli* (Kale et al., 2011), which has a parvulin-like PPIase domain from residues 227 to 357 (Dartigalongue and Raina, 1998). In order to gain evidence for potential PPIase activity for Cj0694, the ability of the protein to accelerate the rate of the proline isomerisation-limited refolding of RCM-RNase T_1_ was examined (Rudd et al., 1995). The refolding of RCM-RNase T_1_ is rate-limited by the prolyl *cis-trans* isomerisation of Pro39 and Pro55, and can be followed by tryptophan fluorescence spectroscopy (Mücke and Schmid, 1992). The two disulphide bonds in RNase T_1_ (Cys2-Cys10 and Cys6-Cys103) are essential in maintaining its conformational stability. Therefore, breaking these bonds results in unfolding of the protein under native conditions. The RCM-RNaseT_1,_ like the native RNaseT_1,_ becomes catalytically active in the presence of 2 M NaCl (Pace et al., 1988). Thus, re-folding of the protein can be enhanced, by increasing the concentration of NaCl. RNase T_1_ has a single tryptophan (Trp59) which is located in a hydrophobic environment in the folded protein (Moors et al., 2009). Refolding of the RCM-RNase T_1_ results in an increase of Trp fluorescence.

The PPIase activity of Cj0694 was demonstrated by monitoring the tryptophan fluorescence of RCM-RNaseT_1_ in the presence of 4 M NaCl. The purified periplasmic chaperone PEB4 was used as a positive control for PPIase activity (Kale et al., 2011). Refolding of RCM-RNaseT_1_ was initiated by a 50-fold dilution of the unfolded protein (stored in the absence of NaCl). Cj0694 or PEB4 were added to the RCM-RNase T_1_ prior to the dilution. As shown in **Figure 4C**, the rate of refolding, as reported by the increase in the steady-state Trp59 fluorescence intensity, is slow in the absence of a PPIase. However, a marked acceleration of the RCM-RNase T_1_ refolding rate was clearly seen in the presence of Cj0694. The activity was dependent on the concentration of Cj0694 and comparable to that determined for PEB4 as a control protein. The data clearly show that Cj0694 has PPIase activity, similar to that of PEB4.

### Cj0694 Has Chaperone Activity With Model Proteins

**FIGURE 5:**
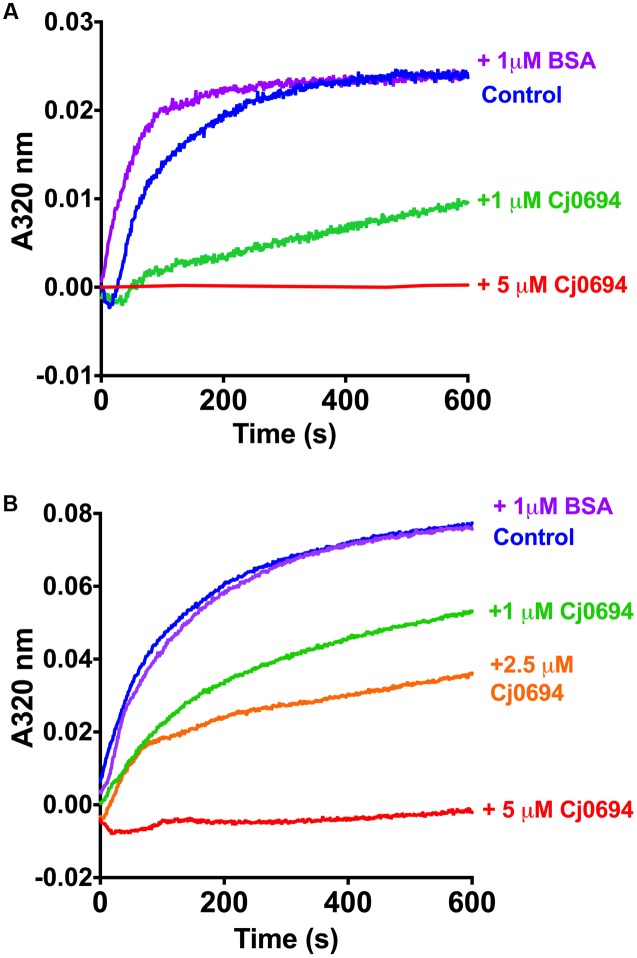
**Inhibition of protein aggregation by Cj0694.** Model proteins Lysozyme **(A)** or Rhodanese **(B)** were unfolded using guanidine-HCl and their aggregation due to renaturation at 25°C monitored in the absence (control) or presence of Cj0694 by light scattering at 320 nm. An additional control contained 1 μM bovine serum albumen (BSA) to ensure that the observed inhibition of aggregation was specifically catalyzed by Cj0694. The traces shown are typical of several experiments performed.

In order examine the chaperone activity of Cj0694, the ability of the protein to inhibit the aggregation of renaturing substrate proteins, measured spectrophotometrically by light scattering, was determined. Two unrelated commercially available model substrate proteins were used; rhodanese and lysozyme. Unfolding of these proteins was carried out as previously described Ideno et al. (2000; see Materials and Methods). Renaturation was initiated by a large dilution of the denatured protein into buffer, to give a final concentration of 1.0 μM, with incubation at 25°C in the absence or presence of Cj0694, with BSA as a negative control. In the absence of Cj0694, the renaturation of either protein resulted in progressive protein aggregation as indicated by an increase in light scattering at 320 nm (**Figure 5**). However, adding Cj0694 in increasing concentrations progressively inhibited the aggregation of both rhodanese and lysosyme as measured by a clear decrease in the light scattering kinetics (**Figure 5**). The control protein BSA added in place of Cj0694 did not inhibit protein aggregation. These results suggest that Cj0694 has chaperone activity that prevents protein aggregation, a role consistent with binding client proteins maintained in only a partially folded state before transfer to the BAM complex for insertion in the OM.

## Discussion

The functioning of the OM requires the correct localization of OMPs catalyzed by the BAM complex and a network of periplasmic chaperone proteins. From previous studies, homology searches and structural comparisons to *E. coli* chaperones, there appear to be five chaperone-like proteins that could play a role in OMP biogenesis in *C. jejuni*: Cj0596 (PEB4), Cj1069 (VirK-like), Cj1228 (HtrA), Cj0694 (PpiD-like) and Cj1289 (SurA-like). We have previously solved the structure of Cj1289 and showed it is indeed a SurA-like enzyme but with only one parvulin domain, while Cj0694 was identified as a likely PpiD homolog but was not further characterized (Kale et al., 2011). In this study, we successfully obtained mutants in *cj0694* and *cj1289* and by comparing their phenotype to *peb4*, *virK* and *htrA* mutants in an isogenic background, we have obtained evidence for their involvement in OM integrity.

Novik et al. (2009) reported the reduced virulence of a *virK* mutant in *C. jejuni* 81–176 in epithelial cells lines and a mouse model, demonstrating its importance as a virulence factor. In *E. coli* VirK is thought to be a periplasmic chaperone for the plasmid-encoded toxin (Pet), an autotransporter produced by enteroaggregative *E. coli* (Tapia-Pastrana et al., 2012). While VirK in *E. coli* is periplasmic, Novik et al. (2009) showed that the *C. jejuni* VirK homolog is associated with the inner membrane on the cytoplasmic face, and so may act as a chaperone prior to Sec-mediated export. We have shown that a *virK* mutant in *C. jejuni* NCTC 11168 displays decreased growth, enhanced motility and biofilm formation, and a strongly hydrophobic cell surface – the latter phenotype shared only with the *peb4* mutant (**Figures 1A–C,E**). This supports the hypothesis that VirK in *C. jejuni* may play a more general role in OM or cell surface biogenesis than reported in *E. coli*, where VirK is necessary, and potentially specific, for Pet toxin secretion (Tapia-Pastrana et al., 2012), a system absent in *C. jejuni.*

Cj1228 in *C. jejuni* is homologous to the *E. coli* high temperature required protein HtrA, formerly DegP, a serine protease with chaperone activity. HtrA has been shown to be essential for *E. coli* survival at high temperatures, and this phenotype has been confirmed in *C. jejuni* (Lipinska et al., 1989; Brøndsted et al., 2005; Boehm et al., 2015). It is known that HtrA is secreted by *C. jejuni* and *H. pylori* in the gut to digest the host cell adhesion protein *E*-cadherin, and recently this was shown to be mediated by OM vesicles (Elmi et al., 2016). However, the reduced viability of *C. jejuni* at high temperatures in the absence of HtrA *in vitro* suggests it plays a role in the cell envelope unrelated to pathogenesis. It has been suggested that HtrA in *E. coli* may function to rescue OMPs that dissociate from the SurA pathway, preventing their aggregation in the periplasm (Sklar et al., 2007). In our work, the *htrA* mutant had a similar growth defect to the other chaperone mutants, but displayed no change in motility, cell surface hydrophobicity or OM integrity compared to wild-type (**Figures 1B,E,F**). However, the *htrA* mutant did show increased biofilm formation and the highest autoagglutination rate of all mutants tested, which could be consistent with a lack of extracellular protease activity.

The remaining chaperones are all related to the *E. coli* SurA protein (Kale et al., 2011). Asakura et al. (2007) and Rathbun et al. (2009) reported a growth defect in a *peb4* mutants made in *C. jejuni* NCTC 11168 and 81-176 respectively, however, the growth defect we found here is much more severe. This may be attributed to the difference in parental strains or growth conditions (42°C in our study vs. 37°C, different microaerobic atmospheres). However, our data do show that a Δ*cj0596* (*peb4*) mutant in NCTC 11168H displays enhanced autoagglutination, motility and biofilm formation (**Figures 1B–D**), similar to the phenotypes found previously for a 81–176 *peb4* mutant (Rathbun et al., 2009; Rathbun and Thompson, 2009), although Asakura et al. (2007) reported lower biofilm forming ability of an NCTC 11168 *peb4* mutant. Of all the mutants, HΔ*cj0596* displayed the greatest growth defect and strongest deficiency in OM integrity (**Figures 1A,F**), supporting a key role for PEB4 in OM biogenesis in *C. jejuni*.

Structurally, Cj1289 more closely resembles SurA than does PEB4 (Kale et al., 2011) and we propose to designate it SalC (SurA-like chaperone). SurA is considered the major periplasmic chaperone in *E. coli*, and it has been shown by differential proteomics that inactivation of *surA* in a *skp* minus background results in diminished levels of nearly all OM β-barrel proteins (Denoncin et al., 2012). The HΔ*cj1289* mutant displayed generally similar phenotypes to the *peb4* mutant, with the exception of a less severe growth defect and a less hydrophobic cell surface (**Figures 1A–F**). If PEB4 and Cj1289 were simply redundant then neither single mutant would be expected to show a strong phenotype unless both were deleted in the same background, as is the case for *skp* and *surA* in *E. coli*, where deletion of both is synthetically lethal (Rizzitello et al., 2001). Given both single mutants showed strong phenotypes relating to OM structure and function, and purified PEB4 and Cj1289 had different folding activities *in vitro* (Kale et al., 2011), we suggest PEB4 and Cj1289 represent the two major periplasmic chaperones in *C. jejuni* that operate as non-redundant pathways for specific client proteins. Further work on the OM protein profile of *peb4* and *cj1289* mutants using a proteomics approach as used by Denoncin et al. (2012) is needed to confirm this hypothesis and to identify specific client proteins.

Cj0694 is predicted to be a periplasmic facing, inner membrane anchored protein most closely resembling PpiD from *E. coli* (Kale et al., 2011). Overexpression of PpiD was able to rescue a lethal *surA skp* double mutant in *E. coli*, and deletion of *ppiD* confers a conditional phenotype on a *htrA* mutant background, suggesting cooperation between *ppiD* and *htrA* as general foldases (Matern et al., 2010). The *cj0694* mutant we constructed had a strong OM integrity phenotype, comparable to that of the *peb4* and *cj1289* mutants (**Figure 1F**), consistent with Cj0694 acting as a chaperone having a significant effect on OM composition. There is clear evidence that in *E. coli*, PpiD interacts with the Sec complex in the inner membrane and participates in folding of newly emerging OM and periplasmic proteins (Sachelaru et al., 2014; Wang et al., 2016). Given the topology, bioinformatics, the pleiotropic phenotype of *cj0694* deletion and Cj0694’s broad substrate range *in vitro* (see below), we suggest Cj0694 is the *C. jejuni* equivalent of PpiD.

**FIGURE 6:**
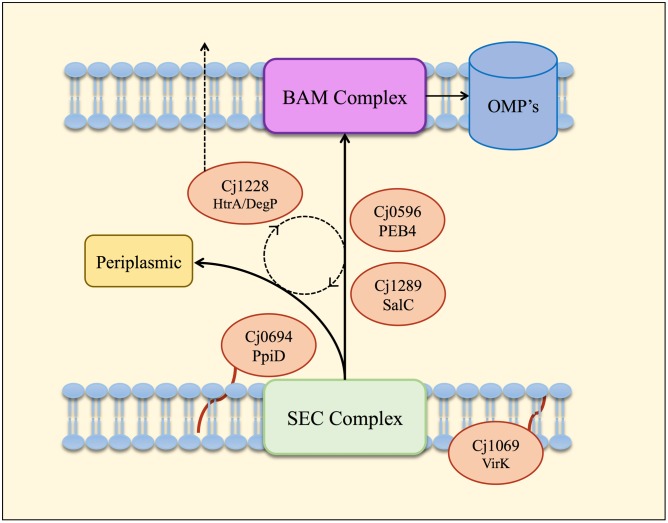
**Model of the periplasmic chaperone network of *C. jejuni*.** OM and periplasmic proteins emerging from the Sec export complex interact with PpiD (Cj0694) for initial folding and translocation to the SalC (Cj1289) or PEB4 (Cj0596) pathway. VirK (Cj1069) may interact with certain substrates in the cytoplasm prior to their entrance into the SEC complex, based on the localization of this protein determined by Novik et al. (2009). SalC and PEB4 are proposed to translocate partially folded OMPs across the periplasm to the BAM complex, where they are inserted into the OM. HtrA (Cj1228) may participate in folding various periplasmic proteins or possibly to rescue OMPs that dissociate from SurA or PEB4 before reaching the OM. HtrA is also secreted from the cell (Hoy et al., 2012) probably mediated by OM vesicles (Elmi et al., 2016).

Overall, the HΔ*cj0694* and HΔ*cj1289* strains displayed a range of similar physiological phenotypes (except autoagglutination) which indicated defects affecting OM structure and function. The highly compromised OM integrity phenotype of HΔ*cj0694* and HΔ*cj1289* was significantly restored by genetic complementation; an incomplete phenotype presumably results from decreased gene expression at the integration locus, even though the native gene promoters were used. Nevertheless, taken together, the mutant and complementation data demonstrate the importance of PpiD and SalC in OM structure and reinforces their role as periplasmic chaperones in *C. jejuni* (**Figure 6**). The 2D-gel results obtained here suggested that a general reduction in OM protein abundance occurred in both the *cj1289* and *cj0694* mutants but we were unable to definitively identify client proteins of the cognate chaperones. Previous 2D-gel studies with *peb4* mutants have identified changes in the expression levels of several proteins compared to the wild-type strain, with decreases in abundance of several OM and periplasmic proteins, including the major outer membrane protein (MOMP), porins (OmpA, Omp50), the haemin OM receptor (CirA), the cysteine binding protein (Cj0982) and the iron receptor (FepA) (Asakura et al., 2007). In addition, Rathbun et al. (2009) found a decrease in the level of three OMPs, the MOMP, the fibronectin binding protein (CadF) and the Omp50 protein (Rathbun et al., 2009; Rathbun and Thompson, 2009). However, a problem with analyzing OM samples of *C. jejuni* is that because the MOMP is such an abundant protein it can make observing much less abundant OMPs very difficult unless the gels are overloaded, which leads to resolution problems. This, combined with the simple protein staining based method used here (and in most other studies) does not allow subtle variations in individual protein abundance to be reliably quantified. Ideally, a method such as SILAC should be applied in future work, where differentially isotopically labeled wild-type and mutant cells can be mixed and processed as a single sample, with mass spectrometry of the proteins allowing accurate abundance ratios to be determined.

The clear phenotypic changes in the *cj0694* mutant discussed above prompted us to examine the biochemical properties of the Cj0694 protein, which we successfully purified in recombinant form in this study. Our results revealed that Cj0694 has an easily demonstrable catalytic activity as a PPIase. Interestingly, despite the similarity between Cj0694 and *E. coli* PpiD discussed above, the latter protein was shown to be devoid of PPIase catalytic activity (Matern et al., 2010; Weininger et al., 2010), and this was also found to be the case for *Yersinia pseudotuberculosis* PpiD (Obi et al., 2011). *E. coli*, PpiD consists of an α-helical transmembrane domain and three periplasmic domains. The first and third domains are proposed to be chaperone domains and the second domain (residues 227 – 357) was identified as a parvulin-like PPIase domain (Dartigalongue and Raina, 1998), which was structurally confirmed by NMR spectroscopy (Weininger et al., 2010). This domain was shown to have high structural similarities to the first parvulin domain of SurA (Weininger et al., 2010) which is known to be inactive as a PPIase (Behrens et al., 2001). The molecular basis of the intriguing difference in the PPIase activity of Cj0694 and PpiD must await structural studies of Cj0694; we have thus far been unsuccessful in obtaining diffracting crystals of this protein. The ability of Cj0694 to act as a chaperone was tested by refolding assays, using the unrelated model proteins lysozyme and rhodanese. It was found that Cj0694 was active in preventing aggregation of both these proteins, in a concentration-dependent manner. This would support the conclusion that Cj0694 has a rather general role in the periplasm as a low specificity chaperone for both periplasmic and OM proteins, which is consistent with work which indicates that *E. coli* PpiD has much lower substrate specificity than SurA (Stymest and Klappa, 2008).

## Conclusion

We have obtained functional and biochemical evidence for a key role for Cj0694 and Cj1289 as periplasmic chaperones acting alongside PEB4 and possibly HtrA in a network (**Figure 6**) that ensures correct OM biogenesis and integrity, properties essential for *C. jejuni* survival and pathogenesis.

## Author Contributions

AT, SZ, and DK designed and executed experiments and analyzed the data. AT and DK wrote the manuscript.

## Conflict of Interest Statement

The authors declare that the research was conducted in the absence of any commercial or financial relationships that could be construed as a potential conflict of interest.

## Footnotes

1 http://matrixscience.com
