# Supplementary material for: The Periplasmic Chaperone Network of Campylobacter jejuni: Evidence that SalC (Cj1289) and PpiD (Cj0694) Are Involved in Maintaining Outer Membrane Integrity
Source: Front Microbiol. 2017 Mar 28;8:531. doi: 10.3389/fmicb.2017.00531 (PMC5368265; doi:10.3389/fmicb.2017.00531)
Supplement: Supplementary file 1 [file Data_Sheet_1.PDF]

Supplementary Material

The periplasmic chaperone network of *Campylobacter jejuni*: Evidence that SalC (Cj1289) and PpiD (Cj0694) are involved in maintaining outer membrane integrity

Aidan J. Taylor[1], Shadi A. Zakai[2] and David J. Kelly[1 *]

[*] Correspondence: Professor David J. Kelly: d.kelly@sheffield.ac.uk

Supplementary Table 1. PCR Primers used in this study. Sequences homologous to the gene to be amplified are shown in lowercase. Adaptors for ISA cloning and restriction sites are shown in UPPERCASE. Random bases added to primers are shown in *italics*.

Primer	Sequence 5' - 3'	Description
KanF	attctccttggttctcatgtttgacagcttat	pJMK30 kanamycin resistance cassette amplification primer, forward
KanR	gcacaccttggctaggtactaaaacaattcat	pJMK30 kanamycin resistance cassette amplification primer, reverse
0596F1	GAGCTCGGTACCCGGGGATCCTCTAGAGTCatgagtatctacagccatga	Mutagenesis primer for *cj0596*, fragment 1 forward
0596R1	AAGCTGTCAAACATGAGAACCAAGGAGAATaatcaaagttgctgcgacta	Mutagenesis primer for *cj0596*, fragment 1 reverse
0596F2	GAATTGTTTTAGTACCTAGCCAAGGTGTGCcaaatttgatgaagtaaaac	Mutagenesis primer for *cj0596*, fragment 2 forward
0596R2	AGAATACTCAAGCTTGCATGCCTGCAGGTCaagatcaagcatatgagaac	Mutagenesis primer for *cj0596*, fragment 2 reverse
0694F1	GAGCTCGGTACCCGGGGATCCTCTAGAGTCgatcttgatcacaagcaatg	Mutagenesis primer for *cj0694*, fragment 1 forward
0694R1	AAGCTGTCAAACATGAGAACCAAGGAGAATgagttaaagcaccattgcta	Mutagenesis primer for *cj0694*, fragment 1 reverse
0694F2	GAATTGTTTTAGTACCTAGCCAAGGTGTGCtaagcaagaacttgttgatg	Mutagenesis primer for *cj0694*, fragment 2 forward
0694R2	AGAATACTCAAGCTTGCATGCCTGCAGGTCcttgtgaaatcactatatgg	Mutagenesis primer for *cj0694*, fragment 2 reverse
1069F1	GAGCTCGGTACCCGGGGATCCTCTAGAGTCtaggacaaggatacaatgac	Mutagenesis primer for *cj1069*, fragment 1 forward
1069R1	AAGCTGTCAAACATGAGAACCAAGGAGAATtctagaagtcttacttgagg	Mutagenesis primer for *cj1069*, fragment 1 reverse
1069F2	GAATTGTTTTAGTACCTAGCCAAGGTGTGCcctagaagaaatcccttcgc	Mutagenesis primer for *cj1069*, fragment 2 forward
1069R2	AGAATACTCAAGCTTGCATGCCTGCAGGTCactaccgctttgtgcataac	Mutagenesis primer for *cj1069*, fragment 2 reverse
1228F1	GAGCTCGGTACCCGGGGATCCTCTAGAGTCcatagaatcgccgtattggt	Mutagenesis primer for *cj1228c*, fragment 1 forward
1228R1	AAGCTGTCAAACATGAGAACCAAGGAGAATattcacacgattagccgttg	Mutagenesis primer for *cj1228c*, fragment 1 reverse
1228F2	GAATTGTTTTAGTACCTAGCCAAGGTGTGCtcataggtgttggacaaagt	Mutagenesis primer for *cj1228c*, fragment 2 forward

1228R2	AGAATACTCAAGCTTGCATGCCTGCAGGTCggtaatcatctgctccaagc	Mutagenesis primer for *cj1228c*, fragment 2 reverse
1289F1	GAGCTCGGTACCCGGGGATCCTCTAGAGTCcgcacaacaggttctatacc	Mutagenesis primer for *cj1289*, fragment 1 forward
1289R1	AAGCTGTCAAACATGAGAACCAAGGAGAATggtatttgcactagccaaac	Mutagenesis primer for *cj1289*, fragment 1 reverse
1289F2	GAATTGTTTTAGTACCTAGCCAAGGTGTGCttcatacaggattattttga	Mutagenesis primer for *cj1289*, fragment 2 forward
1289R2	AGAATACTCAAGCTTGCATGCCTGCAGGTCgctataggatatgaaggtgc	Mutagenesis primer for *cj1289*, fragment 2 reverse
0694pAF	*acac*CAATTGctcttgaacttcattaagtaaaact	Complementation primer for *cj0694*, gene amplification, forward, MfeI adaptor
0694pAR	*acac*TCTAGAtagatttagaagcaagctctatatt	Complementation primer for *cj0694*, gene amplification, reverse, XbaI adaptor
1289pAF	*acac*CAATTGgctttagtagcttctttgga	Complementation primer for *cj1289*, forward, MfeI adaptor
1289pAR	*acac*TCTAGAcgtaataaagctattgcaaag	Complementation primer for *cj1289*, reverse, XbaI adaptor
0694-OEF-pBAD	*aata*CTCGAGgattttaatctcaataga	Forward primer for overexpression of cj0694 in pBAD
0694-OER-pBAD	*aata*GAATTCtcaattccctttataataaat	Reverse primer for overexpression of cj0694 in pBAD

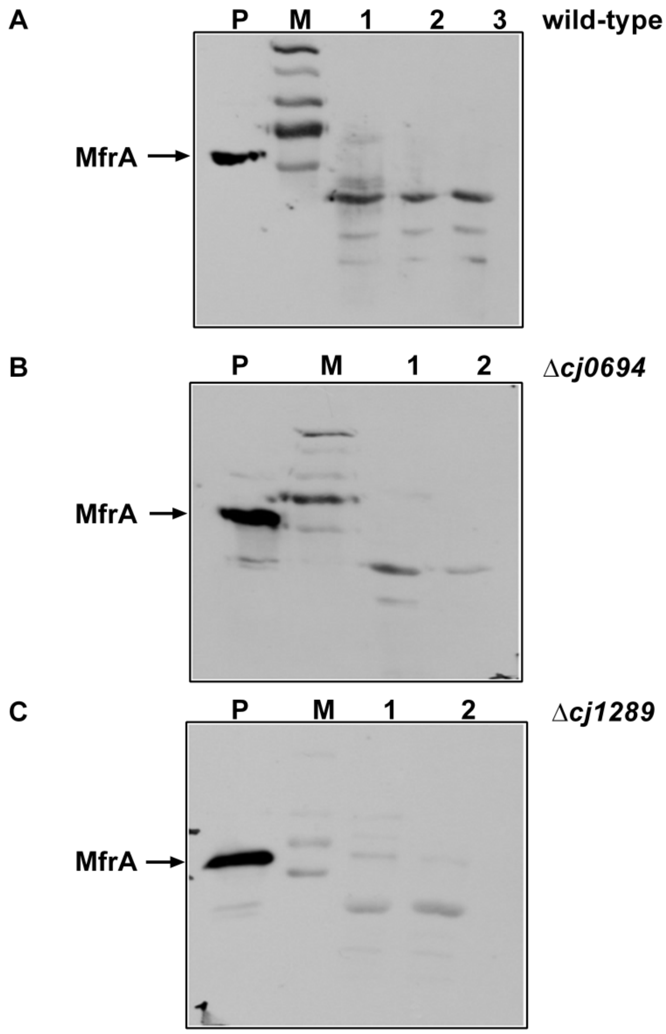

Supplementary Figure 1. MfrA immunoblots to check contamination of OM fractions with periplasm. The ~ 65 kDa MfrA protein in the periplasmic fractions of each strain is shown. Lane (M) shows the PageRuler pre-stained protein markers. Numbered lanes show independently prepared OM fractions of each strain. (A) 11168H wild type (B) HΔ*cj0694* (C) HΔ*cj1289.*
